# Optimising detector readout settings for the detection of spatial correlations between SPDC photon-pairs

**DOI:** 10.1038/s41598-024-84200-x

**Published:** 2025-01-07

**Authors:** K. Roberts, T. Gregory, O. Wolley, M. J. Padgett

**Affiliations:** School of Physics and Astronomy, University of Glasgow, Glasgow, United Kingdom

**Keywords:** Quantum optics, Quantum enhanced imaging, Sensor readout, Quantum optics, Imaging and sensing, Optical sensors

## Abstract

SPDC photon-pairs exhibit spatial correlations which can be measured using detector arrays sensitive to single photons. However, these detector arrays have multiple readout modes and in order to optimise detection it is important to select the optimum mode to detect the correlations against a background of optical and electronic noise. These quantum correlations enable applications in imaging, sensing, communication, and optical processing. Here we compare the measurement of spatial correlations for a broad range of readout modes of an EMCCD camera and attempt to characterise the optimal readout mode for our purposes. This assessment is important for the use of detector arrays of different types for use in quantum, low-light, enhanced resolution, imaging systems.

## Introduction

Quantum enhanced imaging schemes are designed to take advantage of correlations between entangled photon-pairs to improve imaging beyond classical limits^1–3^. Photon-pairs generated by Spontaneous Parametric Down-Conversion (SPDC) are generated by the absorption of a single pump photon leading to the emission of photon-pairs within a non-linear crystal. These photon-pairs obey conservation of energy which results in position correlations in the image plane and anti-correlation in their transverse momentum which results in position anti-correlations in the far-field plane^4^. These spatial correlations have been measured, and their violation of the EPR paradox quantified, using a number of different detector technologies such as EMCCD cameras^5–9^, ICCD cameras^10,11^, SPAD arrays^12–15^, and time-tagging event driven detectors^16,17^. Furthermore, these detector technologies have been applied to perform quantum imaging and encompass a range of improvements including those able to image through background noise and loss^18–25^, improve image resolution^26,27^, ghost imaging^28–31^, sub-shot-noise imaging^32–34^, imaging through scattering media^35,36^, phase imaging^37^, hyperspectral imaging^38^, 3D imaging and ranging^39,40^.

We have previously compared the number of frames required to identify spatial correlations between SPDC photon-pairs for two different detector types; namely an EMCCD detector and a photon number resolving qCMOS detector^41^. In that work, we identified that the EMCCD detector excels in the low illumination regimes and that the photon number resolving detector possesses an advantage for pixel occupancy rate at which multiple photon events are registered per pixel. The advantage of the qCMOS detector is that the low readout noise enables photon number resolution at $\ge 1$ per pixel per frame. In contrast, when run at optimum sensitivity, the EMCCD detector is effectively restricted to 0 or 1 photon in each pixel per frame^42,43^. When using the EMCCD detector, it is also important to ensure that the optimal readout mode is selected to enable the detection of single photons and also allow for the identification of the photon-pairs.

In the work presented here, we utilise an EMCCD camera (Andor iXon Ultra 897) to detect spatially correlated photon-pairs generated by SPDC. The number of frames required to identify the correlation peak with a peak-to-noise ratio $(\text {PTNR}) \ge 5$ is assessed for 60 different readout modes varying pre-amp gain, horizontal-shift speed, and vertical-shift speed readout parameters. Evaluations to determine the optimal readout mode for the detection of characteristics under investigation will enable the enhancements offered by quantum imaging schemes to be maximised, potentially allowing for an increase in the speed at which these quantum technologies are adopted.

## Experimental setup

**Fig. 1:**
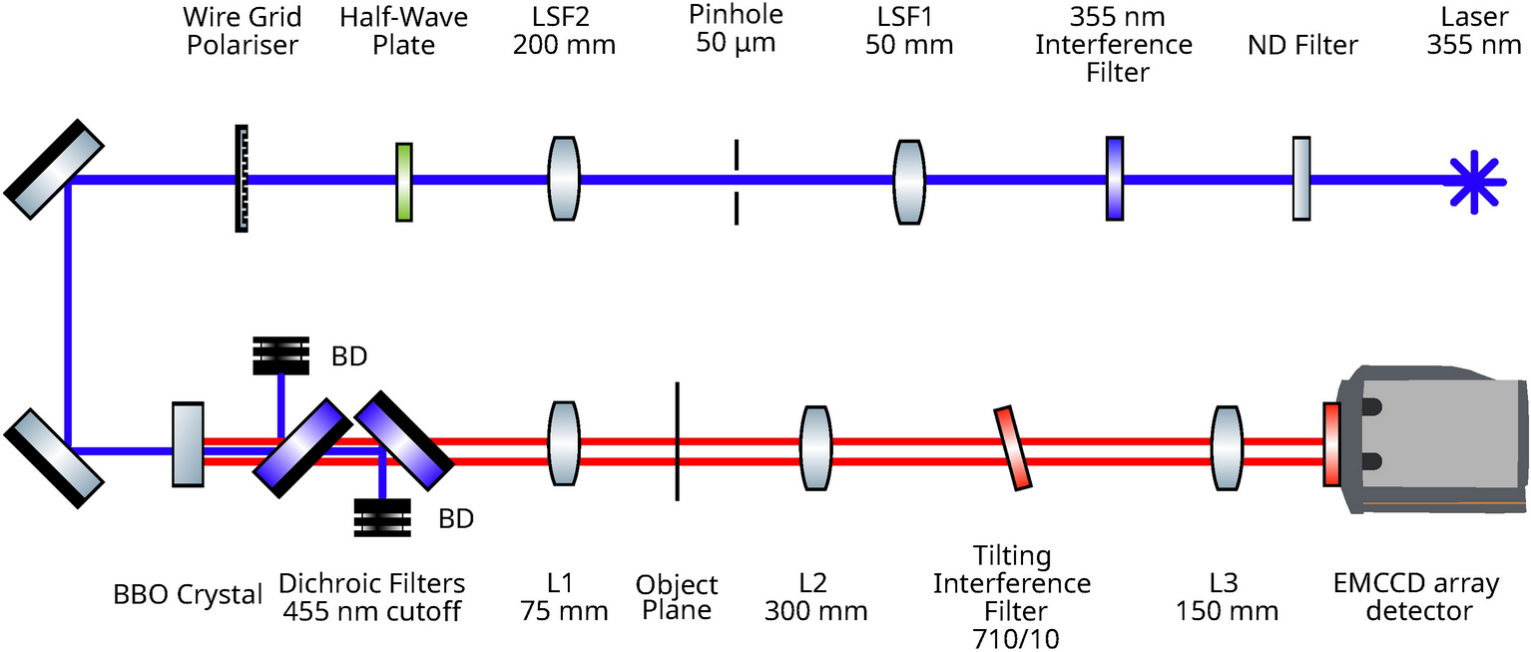
Schematic of the experimental setup. A $355 \text { nm}$ laser pumps a BBO crystal cut for type-II degenerate downconversion to generate downconverted photon-pairs at $710 \text { nm}$. Lenses $L_{1}=75 \text { mm, } L_{2}=300 \text { mm, and } L_{3}=150 \text { mm}$ demagnify the far-field plane of the downconversion crystal by a factor of 2 onto the EMCCD detector.

The experimental setup is as shown in Fig. 1. The pump beam is a laser at $355 \text { nm}$ expanded to $\sim 10 \text { mm}$ in diameter and collimated. The pump beam is then incident upon a BBO crystal of thickness $3 \text { mm}$ cut for type-II parametric downconversion at the degenerate wavelength of $710 \text { nm}$. Immediately after the downconversion crystal, the remaining pump beam is removed by a pair of dichroic mirrors to reduce the autofluorescence of the subsequent optical components so as to minimise their contribution to increased background noise. The downconverted light at $710 \text { nm}$ is selected for by a $710/10 \text { nm}$ interference filter mounted such that it may be tilted to adjust the bandpass, and a second $711/25 \text { nm}$ interference filter is mounted on the detector to exclude both residual pump light and any background light sources. By tilting the first interference filter its bandpass wavelengths may be modified, shifting them to a shorter wavelength and making the filter symmetric about the degenerate wavelength. In the far-field, the effect of tilting the interference filter on the downconversion beams can be observed and used to obtain equally sized collapsed beams, such that the full extent of the detected portions of the downconverted beams contain momentum anticorrelated photon-pairs. In type-II downconversion, the SPDC beams are spatially separated in the far-field of the downconversion crystal, due to their having orthogonal polarisation and the birefringence of the downconversion crystal. The far-field plane of the downconversion crystal is imaged onto the EMCCD detector demagnified by a factor of 2.

### Correlation peak

The strength of transverse momentum correlation is given by Eq. 1 and derived from the transverse momentum uncertainty in the pump and therefore dependent on $w_{p}$ the size of the pump beam, $k_{p}$ the wavenumber of the pump beam, and *f* the focal length of the lens used to Fourier transform from the plane of the crystal to the plane of the detector^44–46^. The detected width of the correlation peak in the far-field may be larger than this theoretically calculated size due to the limited numerical aperture of the optical system, defocus effects, and optical aberrations such astigmatism resulting from the inclined filters. For our experimental configuration, the width of the correlation peak on the camera was observed to be $\sigma _{x} = 2.37$, $\sigma _{y} = 1.37$ pixels.

1 $$\begin{aligned} \sigma _{p_{1} + p_{2}} = \frac{4 f}{k_{p} w_{p}} \end{aligned}$$

### Readout options

The detector used in this experiment is an Andor iXon Ultra 897. The impacts of the different readout parameters will be briefly detailed from the hardware manual such that the impact of changing modes might be considered in our analysis^47^. Vertical shift speed is the time in $\mu \text {s}$ to shift a row of pixels down towards the shift register. Faster vertical shift speeds result in lower Clock Induced Charge (CIC) but reduced efficiency of charge transfer. This reduced efficiency can be compensated for by adjusting the vertical clock amplitude but this results in increased CIC and so the range of vertical clock amplitudes are not considered here. Horizontal shift speed is the rate in $\text {MHz}$ at which the pixels are read from the shift register into the gain register. Lower speeds exhibit reduced readout noise but reduce the overall frame-rate of the camera and total exposure time. The choice of readout modes also impacts the dynamic range of the detector but as the EMCCD cameras are operated under low illumination conditions in our SPDC experiments we do not need to consider these effects. Our choice of readout parameters in our previous works was based upon choosing a low standard deviation in dark frames readout noise and balancing this with an overall readout speed in order to maintain throughput. The read-out time and therefore throughput is dependent mostly on the horizontal shift speed with values of 0.02 s for 17 MHz, 0.03 s for 10 MHz, 0.06 s for 5 MHz, 0.3 s for 1 MHz.

## Results

As in our previous work^41^ we identify the presence of the correlation peak by assessing a peak to noise ratio (${\text {PTNR}}$) on the temporally subtracted correlation peak and defining visibility at a value of ${\text {PTNR}} \ge 5$ within a radius of 3 pixels of the centre pixel. We apply low-pass filter ($\sigma = 0.75$) so as to prevent noise spikes from being identified as the correlation peak incorrectly. We then calculate the correlation peaks calculated over 10, 000 frames for each of the combinations of vertical shift (0.3 μs, 0.5 μs, 0.9 μs, 1.7 μs, 3.3 μs), horizontal shift (17 MHz, 10 MHz, 5 MHz, 1 MHz), and pre-amp gain ($1 \text {, } 2 \text {, }3$) for a fixed EM gain of 1000 and vertical amplitude 0 V for a total of 60 different readout modes.

### High pixel occupancy rate illumination regime

**Fig. 2:**
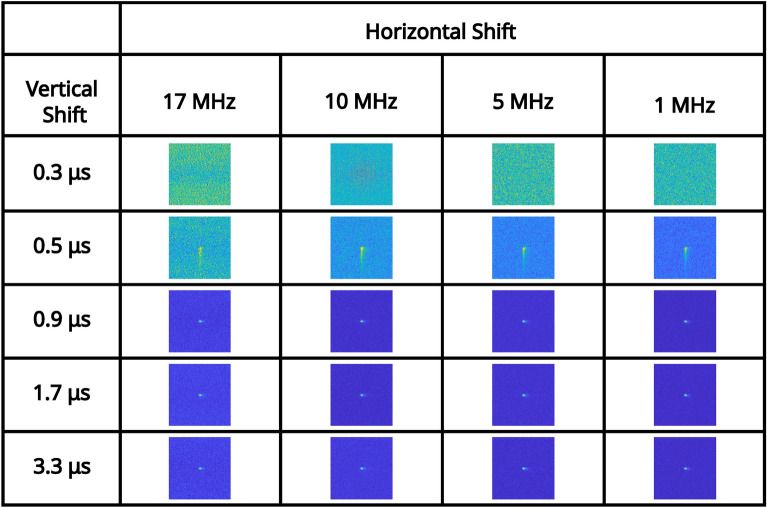
High pixel occupancy rate illumination regime: correlation peak array. A series of correlation peaks acquired in the high pixel occupancy rate illumination regime. Each correlation peak is calculated using 10, 000 camera frames for the subset of combinations of readout modes acquired with a pre-amp gain of 1. The vertical shift speed decreases by row and the horizontal shift speed decreases by column. The camera frames used to generate the correlation peaks are presented as their sum in Fig. 3. The x and y axes represent displacement in the cross-correlation.

**Fig. 3:**
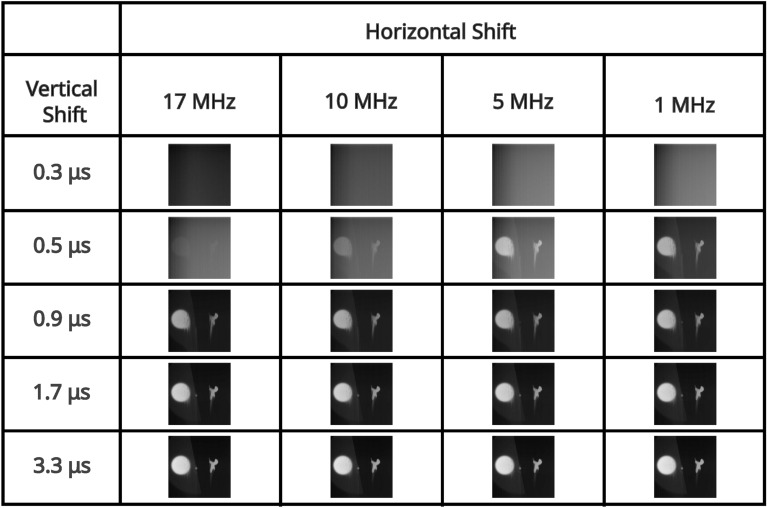
High pixel occupancy rate illumination regime: sum of frames array. A series of full-frame images acquired in the high SPDC illumination regime by summing the analogue output of 10, 000 frames for the subset of combinations of readout modes acquired with a pre-amp gain of 1. The vertical shift speed decreases by row and the horizontal shift speed decreases by column. The x and y axes correspond to pixels on the detector.

For an illumination level that equates to $\sim 0.15$ events per pixel per frame (measured in the readout mode of; vertical shift 1.7 μs, horizontal shift $5 \text { MHz}$, pre-amp gain 1, and EM gain 1000), a series of correlation peaks calculated over 10, 000 frames for each of the combinations of readout modes acquired with a pre-amp gain of 1 are presented in Fig. 2. The pixel occupancy rate for this illumination level for each of the readout modes for pre-amp gain of 1 are shown in Table 1. It can be seen that for vertical shift 0.3 μs the correlation peak on the first row of Fig. 2 and image of the total sum of frames on the first row of Fig. 3 are not visible above the noise level. This is due to charge transfer being ineffective for a vertical clock amplitude of 0 V for this vertical shift speed. For vertical shift of 0.5 μs the correlation peak on the second row of Fig. 2 and image of the total sum of frames on the second row of Fig. 3 are visible but exhibit the effects of charge smearing which is a consequence of a vertical clock amplitude of 0 V.

**Fig. 4:**
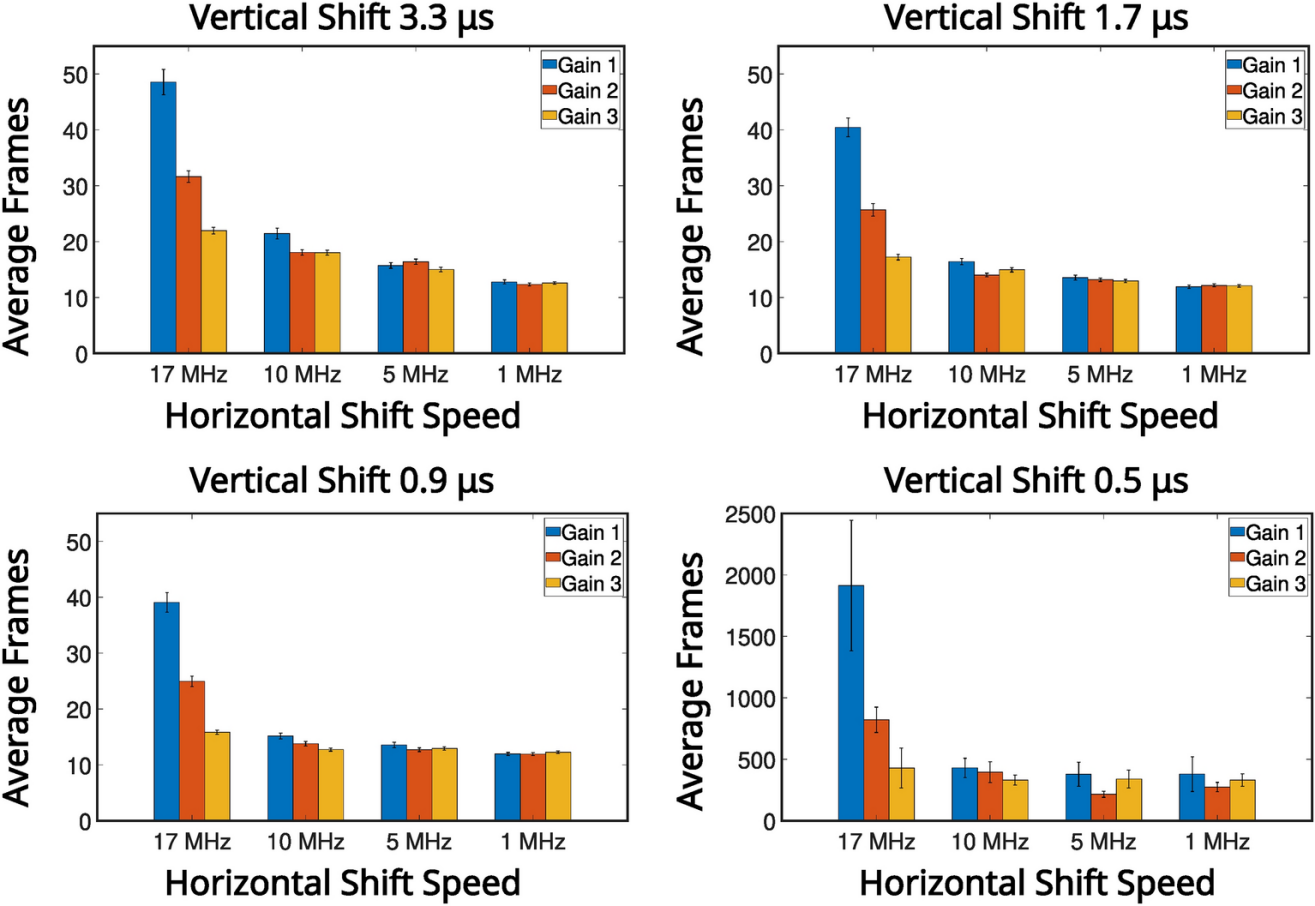
High pixel occupancy rate illumination regime: average number of frames to meet the $\text {PTNR}$ condition for different noise modes. The average number of frames required to meet the $\text {PTNR}$ condition for different noise modes acquired in the high SPDC illumination regime spilt by vertical shift speeds. The values are calculated from 10, 000 frames per readout mode.

In Fig. 4 the average number of frames required to satisfy the $\text {PTNR} \ge 5$ condition is presented for vertical shifts of 0.9 μs, 1.7 μs, 3.3 μs, horizontal shifts of 17 MHz, 10 MHz, 5 MHz, 1 MHz, and pre-amp gain $1 \text {, } 2 \text {, }3$ are presented in a series of bar charts. It can be seen from the data presented in Fig. 4 that for combinations that equate to slower readout (vertical shifts of 0.9 μs, 1.7 μs, 3.3 μs, and horizontal shifts of 10 MHz, 5 MHz, 1 MHz), that the number of frames required to meet the $\text {PTNR}$ condition is of order $\sim 10-20$ frames. For these vertical readout speeds the horizontal shift speed of $17 \text { MHz}$ requires between around $\sim 15-45$ frames to satisfy the $\text {PTNR}$ condition so around $2\times$ the number of frames required for the slower horizontal shift readout speeds. It is for this horizontal shift speed of $17 \text {MHz}$ also that the effects of different pre-amp gain modes becomes apparent with pre-amp gain of 1 requiring almost twice as many frames compared to pre-amp gain modes 2 and 3. For a vertical shift speed of $0.5 \mu \text {s}$ there is a large increase in the number of frames required to meet the $\text {PTNR}$ condition. This increase in the number of frames required is as might be expected from the smearing present on the correlation peaks in the second row of Fig. 2 which is a result of reduced charge transfer efficiency. For 10 MHz, 5 MHz, 1 MHz ($\sim 200-300$) frames on average are required to meet the $\text {PTNR}$ condition across the different pre-amp gain modes. For $17 \text { MHz}$ $\sim 500-2000$ frames on average are required to determine the presence of a correlation peak depending on the pre-amp gain mode. The effects of readout noise due to reducing the horizontal shifts are evident in the bar charts presented in Fig. 4, however, the effects of increased CIC as a consequence of reducing the vertical shift speed are less pronounced.

**Table 1:** High pixel occupancy rate illumination regime: Binarised pixel pixel occupancy rate. This table presents the pixel occupancy rate and occupancy rate to dark noise ratio as measured by binarising at a value of $\mu + 3 \sigma$ analogue counts for the data to calculate the correlation peaks presented Fig. 2.

		Horizontal shift			
		17 MHz	10 MHz	5 MHz	1 MHz
Vertical shift	0.3 μs	0.0002	0.0008	0.0013	0.0040
		$1.0574\times$	$1.0150\times$	$1.0000\times$	$0.9692\times$
	0.5 μs	0.0129	0.0537	0.1247	0.5779
		$36.3553\times$	$39.6709\times$	$58.8373\times$	$95.4952\times$
	0.9 μs	0.0146	0.0566	0.1311	0.5927
		$21.9671\times$	$26.8060\times$	$41.5489\times$	$79.8994\times$
	1.7 μs	0.0163	0.0586	0.1343	0.5863
		$11.9827\times$	$15.0268\times$	$25.0465\times$	$57.2255\times$
	3.3 μs	0.0203	0.0663	0.1442	0.5957
		$5.9292\times$	$7.3158\times$	$12.1792\times$	$32.8983\times$

### Low pixel occupancy rate illumination regime

**Fig. 5:**
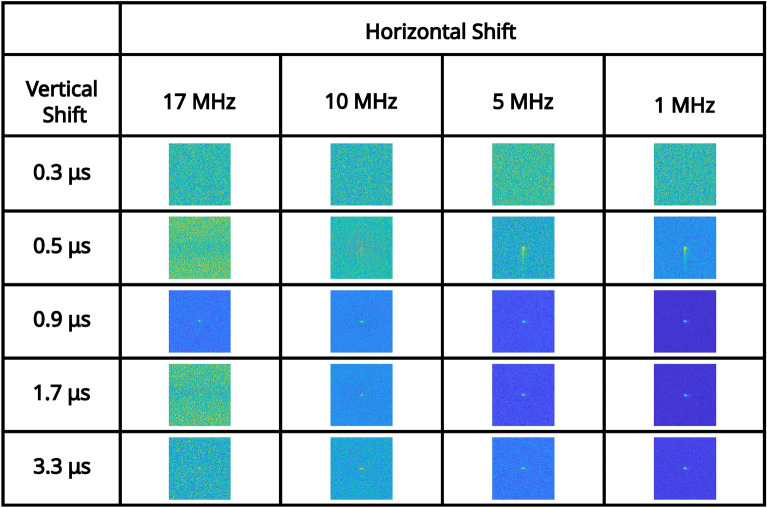
Low pixel occupancy rate illumination regime: correlation peak array. A series of correlation peaks obtained using analogue output data from frames acquired in the low pixel occupancy rate illumination regime. Each correlation peak is calculated using 10, 000 camera frames for the subset of combinations of readout modes acquired with a pre-amp gain of 1. The vertical shift speed decreases by row and the horizontal shift speed decreases by column. The camera frames used to generate the correlation peaks are presented as their sum in Fig. 6. The x and y axes represent displacement in the cross-correlation.

**Fig. 6:**
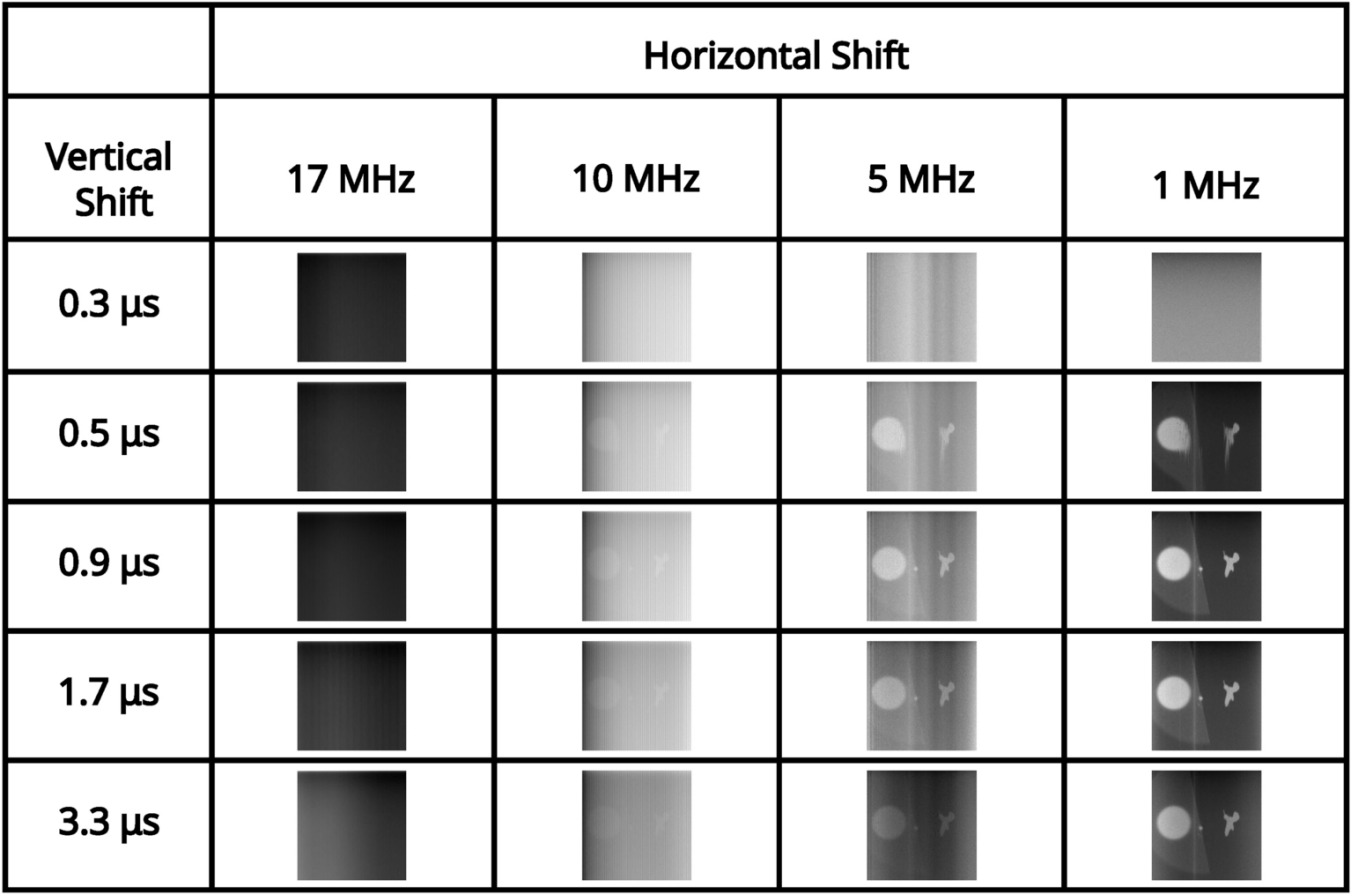
Low pixel occupancy rate illumination regime: sum of frames array. A series of full-frame images acquired in the low SPDC illumination regime by summing the analogue output of 10, 000 frames for the subset of combinations of readout modes acquired with a pre-amp gain of 1. The vertical shift speed decreases by row and the horizontal shift speed decreases by column. The x and y axes correspond to pixels on the detector.

For an illumination level at which the number of photon events is close to equivalent to detector noise events (determined in the readout mode of vertical shift $1.7 \mu \text {s}$, horizontal shift $5 \text { MHz}$, pre-amp gain 1, and EM gain 1000), a series of correlation peaks calculated over 10, 000 frames for each of the combinations of readout modes for pre-amp gain of 1 are presented in Fig. 5. pixel occupancy rate and ratios of binarised illumination to detector noise for this illumination level for each of the readout modes for pre-amp gain of 1 are shown in Table 2. It can be seen again that for vertical shift 0.3 μs there is readout noise such that the correlation peak on the first row of Fig. 5 and image of the total sum of frames on the first row of Fig. 6 are not visible and only noise appears to be present. This is again due to charge transfer being ineffective for a vertical clock amplitude of $0 \text { V}$ for this vertical shift speed. For further fast combinations of readout modes that include a vertical shift of 0.5 μs the correlation peaks are visible for slower horizontal shifts of $5 \text { MHz}$ and $1 \text { MHz}$ but exhibit the effects of charge smearing which is a consequence of a vertical clock amplitude of $0 \text { V}$. For a horizontal shift $17 \text { MHz}$ there is also increased readout noise and the correlation peaks on the second row of Fig. 5 are either not visible, smeared, or barely visible. The reduced visibility of the correlation peak is to be expected because there is a lower proportion of the total analogue counts per frame correspond to correlated photon-pair events. The volume of the correlation peak is twice the number of pair events $N_{p}$^48^ and so with fewer photon-pairs, there is increased relative shot-noise in the subtracted correlation peaks.

**Fig. 7:**
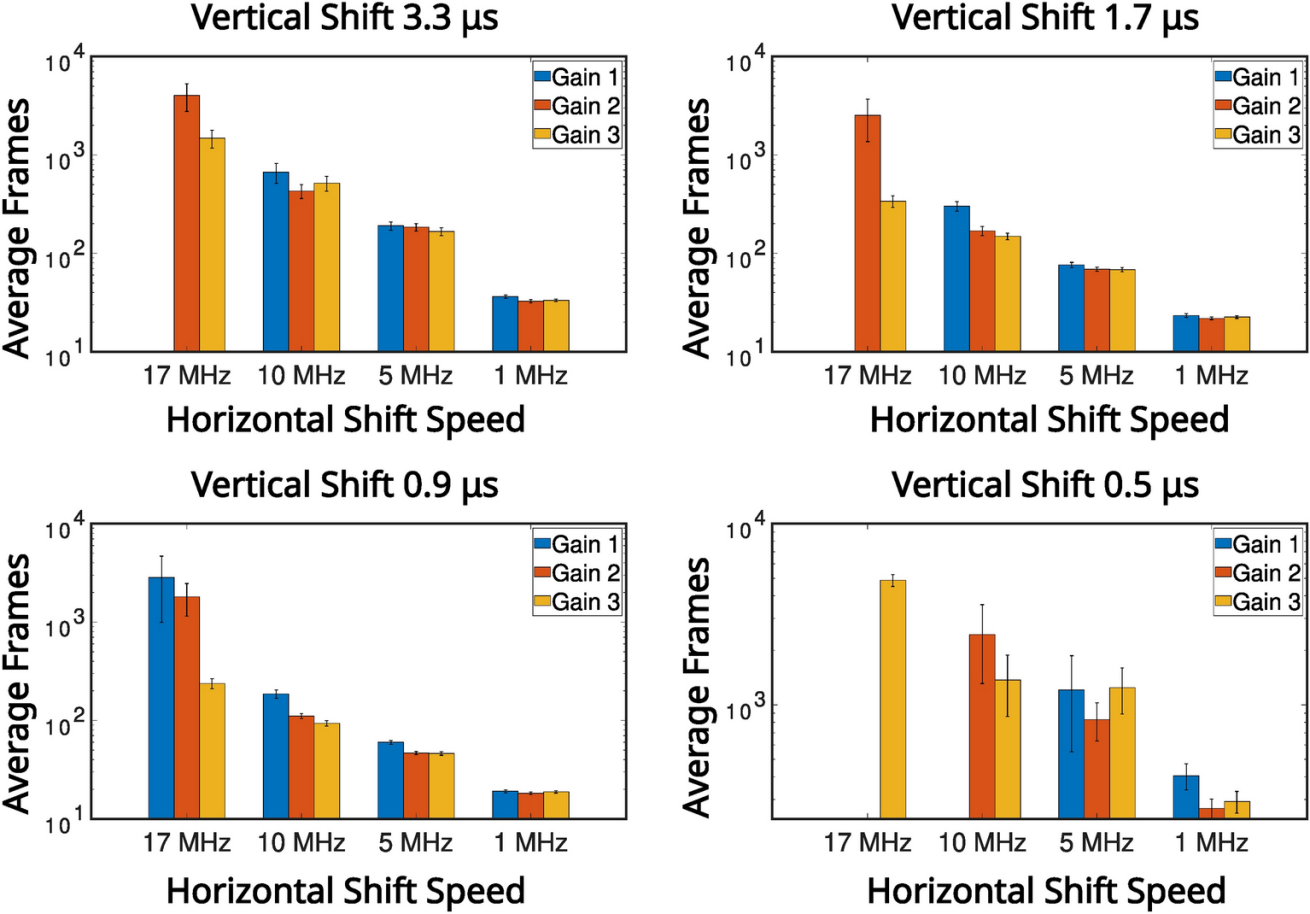
Low pixel occupancy rate illumination regime: average number of frames to meet the $\text {PTNR}$ condition for different noise modes. The average number of frames required to meet the $\text {PTNR}$ condition in the low pixel occupancy rate illumination regime for different noise modes spilt by vertical shift speeds. The values are calculated from 10, 000 frames per readout mode.

In Fig. 7 the average number of frames required to satisfy the $\text {PTNR} \ge 5$ condition is presented for vertical shifts of (0.5 μs, 0.9 μs, 1.7 μs, 3.3 μs, horizontal shifts of 17 MHz, 10 MHz, 5 MHz, 1 MHz, and pre-amp gain ($1 \text {, } 2 \text {, }3$) are presented in a series of bar charts. For a horizontal shift of $17 \text { MHz}$ for each of the presented vertical shifts and for a vertical shift of 0.3 μs for each of the presented horizontal shifts, few peaks were identified per mode combination as shown in Fig. 7. The dearth of identifiable correlation peaks for these readout modes is displayed in Fig. 5. This is with the apparent exception of $17 \text { MHz}$ horizontal shift 0.9 μs vertical shift which required 3000 frames on average to meet the $\text {PTNR}$ condition.

The data presented in Fig. 7 shows that for slower readout speeds (vertical shifts of 0.9 μs, 1.7 μs, 3.3 μs) readouts at $10 \text { MHz}$ require up to $\sim 200-700$ frames, readouts at $5 \text { MHz}$ require $\sim 50-200$ frames, and readouts at $1 \text { MHz}$ require $\sim 20-40$ frames to meet the $\text {PTNR}$ condition. Vertical shift speed of $0.5 \mu \text {s}$ require an increased number of frames for each mode to determine the presence of a correlation peak when compared to other readout modes presented here as might be expected from the smearing present on the correlation peaks in the second row of Fig. 5. For each vertical shift speed there is a decrease in the number of frames required as the horizontal shift speed is reduced. This is expected because the readout noise reduces with reduced readout speed, in addition to more photon events being collected per frame. The effects of reducing the vertical shift speed from 0.9 μs to 1.7 μs to 3.3 μs is more evident in the low pixel occupancy rate illumination regime due to the increased proportion of detector events that correspond to CIC than for the high pixel occupancy rate illumination regime. Therefore, the number of frames required to meet the $\text {PTNR}$ condition is increased in this low pixel occupancy illumination regime.

**Table 2:** Low pixel occupancy rate illumination regime: Binarised pixel pixel occupancy rate. This table presents the pixel occupancy rate and occupancy rate to dark noise ratio as measured by binarising at a value of $\mu + 3 \sigma$ analogue counts for the data to calculate the correlation peaks presented Fig. 5.

		Horizontal shift			
		17 MHz	10 MHz	5 MHz	1 MHz
Vertical shift	0.3 μs	0.0002	0.0007	0.0013	0.0045
		$1.0079\times$	$0.9735\times$	$1.0061\times$	$1.0769\times$
	0.5 μs	0.0008	0.0035	0.0073	0.0395
		$2.3900\times$	$2.6001\times$	$3.5043\times$	$6.7627\times$
	0.9 μs	0.0011	0.0041	0.0083	0.0417
		$1.7223\times$	$1.9911\times$	$2.6901\times$	$5.8215\times$
	1.7 μs	0.0018	0.0055	0.0102	0.0431
		$1.3092\times$	$1.4427\times$	$1.9456\times$	$4.4360\times$
	3.3 μs	0.0042	0.0112	0.0175	0.0524
		$1.2365\times$	$1.2466\times$	$1.5044\times$	$2.9921\times$

### Low pixel occupancy rate illumination regime: binarised frames

**Fig. 8:**
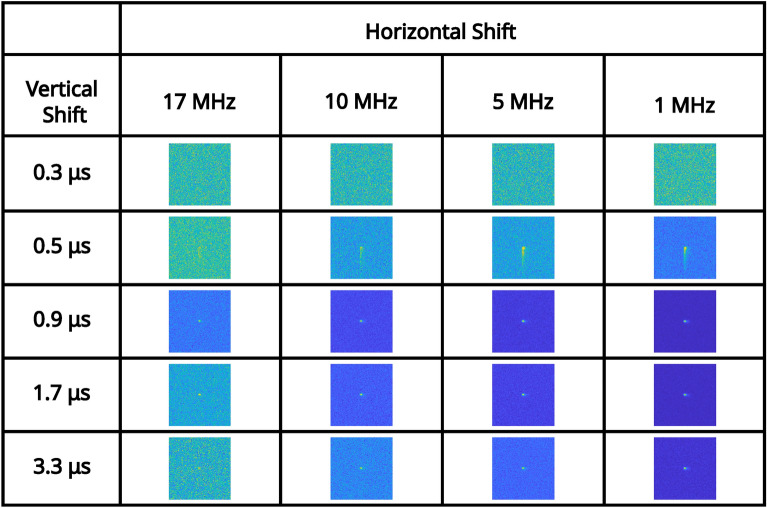
Binarised low pixel occupancy rate illumination regime: correlation peak array. A series of correlation peaks obtained from binarised frames acquired in the low pixel occupancy rate illumination regime. Each correlation peak is calculated using 10, 000 camera frames for the subset of combinations of readout modes acquired with a pre-amp gain of 1. The vertical shift speed decreases by row and the horizontal shift speed decreases by column. The camera frames used to generate the correlation peaks are presented as their sum in Fig. 10. The x and y axes represent displacement in the cross-correlation.

**Fig. 9:**
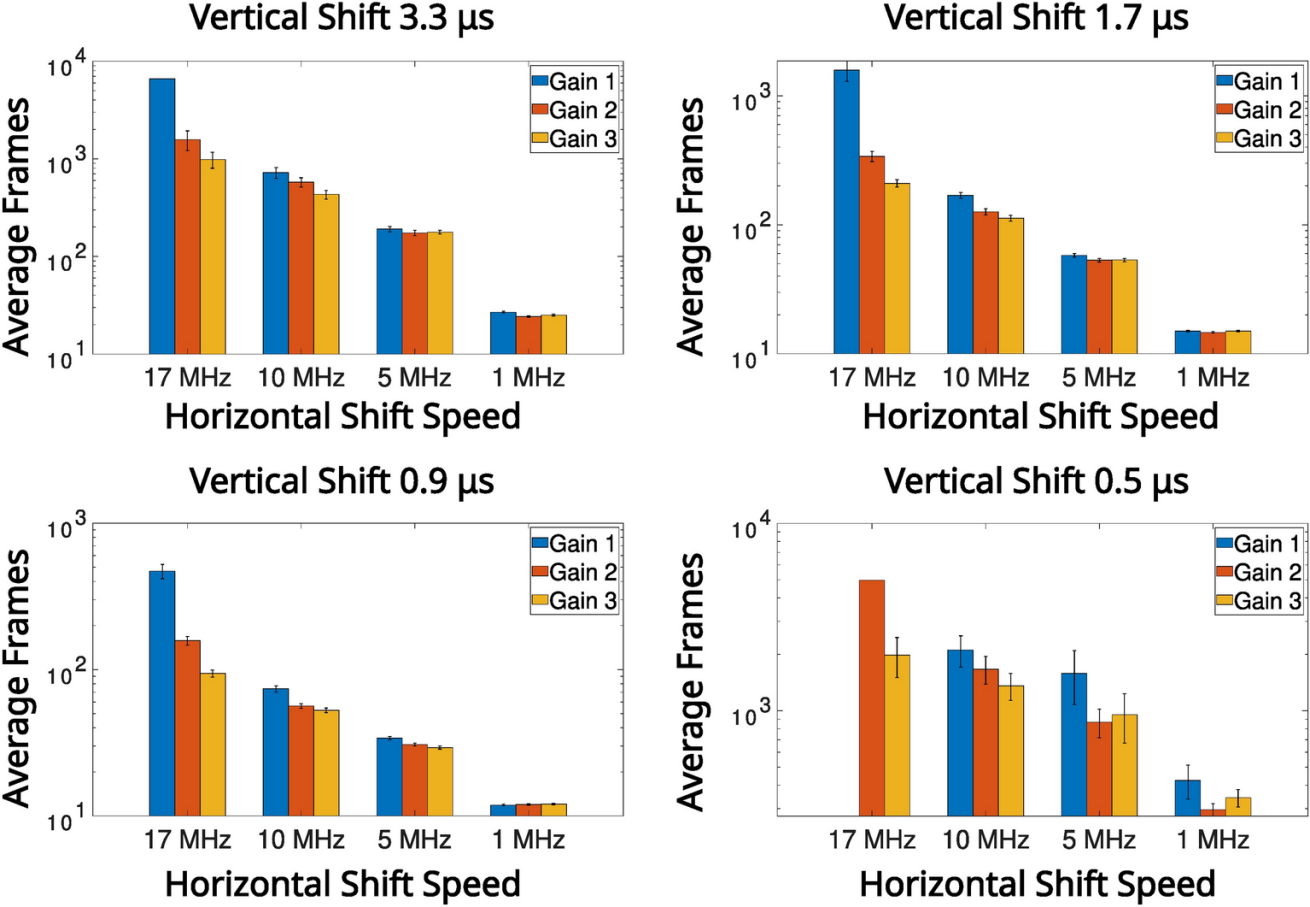
Binarised low pixel occupancy rate illumination regime: average number of frames to meet the $\text {PTNR}$ condition for different noise modes. The average number of frames required to meet the $\text {PTNR}$ condition for different noise modes for binarised frames acquired in the low pixel occupancy rate illumination regime spilt by vertical shift speeds. The values are calculated from 10, 000 frames per readout mode.

**Fig. 10:**
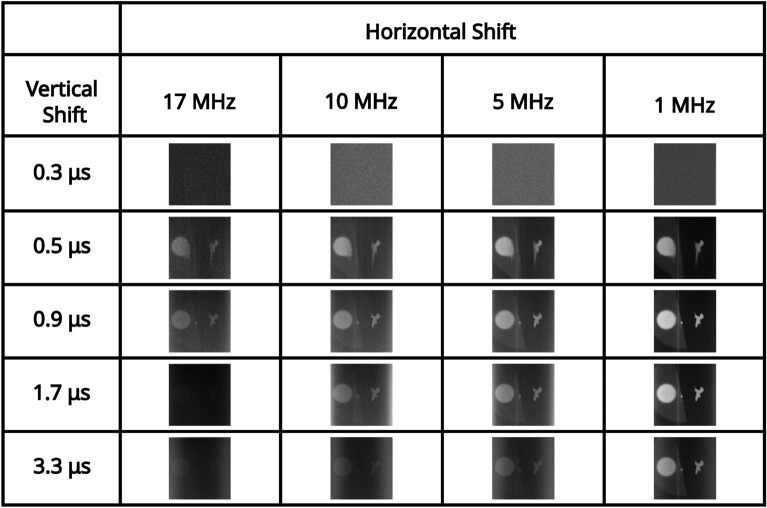
Binarised low pixel occupancy rate illumination regime: sum of frames array. A series of full-frame images acquired in the low SPDC illumination regime by summing the binarised output (at an analogue value of $\mu + 3 \sigma$ analogue counts) of 10, 000 frames for a subset of the combinations of readout modes pre-amp gain of 1. The vertical shift speed decreases by row and the horizontal shift speed decreases by column. The x and y axes correspond to pixels on the detector.

We now apply the same analysis to binarised frames obtained under the low pixel occupancy rate illumination regime to assess whether there is a similar trend as we would expect given our previous work^41^ in which we reported that binarised EMCCD data has an advantage over analogue data in terms of the average number of frames required to meet the $\text {PTNR}$ condition for pixel occupancy rates of $<0.5$ events per pixel per frame. The correlation peaks calculated using binarised frames shown in Fig. 8 present a similar form and trend across the different combinations of readout modes as those for the analogue data presented in Fig. 5. For the binarised data presented in Fig. 9 on average $\sim 0.7\times$ the number of frames on average are required to identify the presence of a correlation peak when compared to that for the analogue data presented in the preceding section. It can also be seen in Fig. 10 that binarising the images cleans up the fixed pattern noise, streaking, and charge smearing present for the average sum of frames when compared to those for non-binarised data in the low pixel occupancy rate illumination regime in the preceding section.

**Fig. 11:**
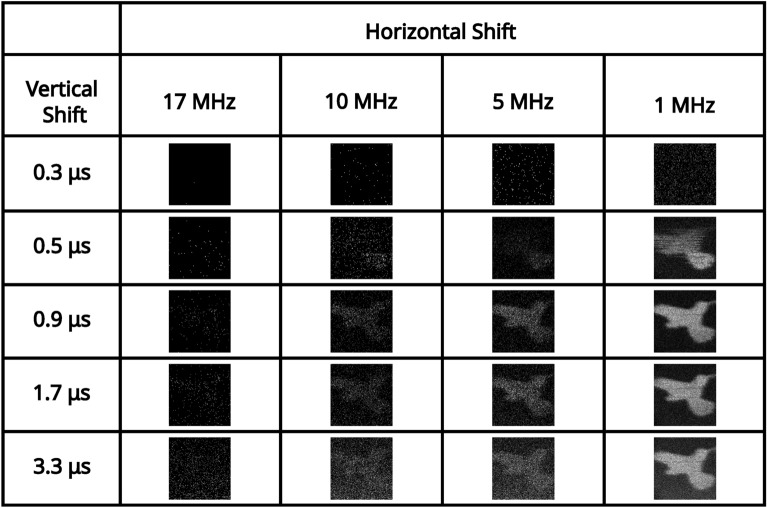
Binarised low pixel occupancy rate illumination regime: AND images array. A series of AND-images acquired by performing the AND-operation to select spatially correlated photon-pairs in binarised frames obtained in the low pixel occupancy rate illumination regime. The images are the result of performing the AND-operation on 10, 000 frames and summing the output. This data represents the subset of combinations of readout modes acquired with a pre-amp gain of 1. The vertical shift speed decreases by row and the horizontal shift speed decreases by column. The x and y axes correspond to pixels on the detector.

For our experiments detecting single photon-events and identifying photon-pairs is necessary to realise the advantages of using a SPDC source. As such, we assess the resulting contrast for the AND-images calculated from the 10, 000 acquired frames for each of the modes that are presented in Fig. 11 and present the image contrast in Table 3. Due to the low number of frames acquired and the resulting low number of AND-events overall, we use AND-images as opposed to the temporally subtracted AND-images here. Image contrast is assessed for the quantum illuminated bird regions of the image with respect to the background. For the data presented in Fig. 11 this contrast metric is maximised for a pre-amp gain of 1 using settings of vertical shift 0.9 μs and a horizontal shift $1 \text { MHz}$. Across all of the combinations tested, the optimal balance of resulting AND-image contrast, rejection of additional readout noise, and speed of acquisition would in fact appear to be for a vertical shift of 0.9 μs, a horizontal shift of $5 \text { MHz}$, pre-amp gain of 1, and EM gain 1000. This readout setting differs from that which we used in our previous work which measured using the readout settings; vertical shift 1.7 μs, horizontal shift $5 \text { MHz}$, pre-amp gain 1, and EM gain 1000. Our previous criteria selected for a low standard deviation in the output analogue counts calculated from dark frames in addition to rejecting modes that feature charge smearing and fixed pattern noise. By using the method presented in this work we have found a mode that results in an improved image assessment metric in the form of resulting AND-image contrast, also whilst maintaining the same data throughput as we had previously.

**Table 3:** Binarised low pixel occupancy rate illumination regime: AND-image contrast. Calculated contrast between the quantum illuminated bird and the background for the series of AND-images acquired using binarised frames in the low pixel occupancy rate illumination regime summed over 10, 000 frames for a subset of the combinations of readout modes acquired with a pre-amp gain of 1 presented in Fig. 11.

		Horizontal shift			
		17 MHz	10 MHz	5 MHz	1 MHz
Verticall shift	0.3 μs	–	–	–	–
	0.5 μs	0.42	0.37	0.45	0.51
	0.9 μs	0.67	0.59	0.65	0.69
	1.7 μs	0.41	0.43	0.52	0.62
	3.3 μs	0.10	0.15	0.23	0.47

## Discussion

We compared the different readout modes for an EMCCD detector when used to observe spatial correlations and we found that many of the modes require a similar number of frames to meet our visibility criterion. For the detector used in this work the performance of the camera was most affected by the faster shift speeds being more sensitive to the choice of pre-amplifier gain mode but for slower readout modes $<17 \text { MHz}$ horizontal shift and $>0.9 \upmu \text {s}$ vertical shift combinations of readout all require of order 10 frames to satisfy the $\text {PTNR} \ge 5$ condition in the high pixel occupancy rate illumination regime. The average number of frames required to meet the $\text {PTNR} \ge 5$ condition is greater in the low pixel occupancy rate illumination regime due to the photon events comprising a reduced proportion of the overall detector readout that also includes sensor noises. This leads to the effects of sensor noise becoming evident as demonstrated by the presence of fixed pattern noises in Fig. 6. The effect of these noises can be mitigated by then binarising the frames as shown in Fig. 10. The effects of binarisation results in a reduction by a factor of $0.7 \times$ in the number of frames required to meet the $\text {PTNR} \ge 5$ condition. These results indicate that when conducting such an experiment it is best to initially deselect readout modes that are either completely incompatible with the experiment or inefficient for the purposes of identifying the detector events required. A combination of other constraints might better define the optimal readout modes of the detector, for example the readout speed restricts the frame rate and therefore places a limit on the timescales of processes that might be observed and also limits the data throughput of the system as is the case for the $1 \text { MHz}$ readout modes presented in this work. Using the method presented here to optimise detector readout settings based on the targeted imaging assessment metric in the illumination regime in which we typically operate our experiments has enabled an improvement in said metric when compared to our previously used readout settings. Previously, the detector readout setting was selected based on a relatively low standard deviation in the analogue counts for dark frames, in addition to rejecting modes that feature charge smearing and fixed pattern noise. Optimising for these seemingly sensible criteria did not necessarily optimise for single photon detection of photon-pairs and the resulting AND-image contrast. In optimising for the experimental assessment metric, in the form of resulting AND-image contrast, we were able to achieve an improvement whilst also maintaining the same data throughput as we had previously.

The detector has a quantum detection efficiency that varies with wavelength. As such, the wavelength used and the subsequent event rate at the detector will affect the number of frames required to acquire a correlation peak and meet the PTNR condition. For example, the quantum efficiency of our Andor iXon Ultra 897 EMCCD detector with a back-illuminated sensor with fringe suppression and dual anti-reflection coated sensor (#EXF) is stated at $810 \text { nm}$ to be $\sim 75\%$, and at $710 \text { nm}$ to be $\sim 90\%$. Due to the lower quantum efficiency at $810 \text { nm}$ we would expect that the number of frames required to meet the PTNR condition increases due to a reduced photon detection efficiency and therefore lower photon-pair coincidence rate, but for the relative differences between the different readout modes to hold. We hope that the approach demonstrated here can help inform researchers to perform initial tests that are directly related to the effects that they are trying to measure in order to identify optimal detector readout parameters prior to conducting their experiments. This method of selecting readout parameters could find use in the case of detectors with an increased number of readout parameters or those that are continuously adjustable.

## Materials and methods

Our experiential procedure is similar to that reported in Roberts et al.^41^ and is summarised here. The laser source used was a JDSU xCyte CY-355-150 Nd:YAG laser with quasicontinuous output at $355 \text { nm}$ with power output at $150 \text { mW}$, a pulse repetition of $100 \pm 10 \text { Mhz}$, and a pulse width of $>10 \text { ps}$. The spatial filter used to expand and collimate the pump beam comprises of a $50 \text { mm}$ lens, a 50 μm pinhole, and a $200 \text { mm}$ lens. The downconversion source was a BBO non-linear crystal cut for type-II downconversion at degenerate wavelength of $710 \text { nm}$. Downconversion crystal dimensions $10 \text { mm } \times 10 \text { mm } \times 3 \text { mm}$. The EMCCD camera used was an Andor Ultra 897 of pixel size 16 × 16 μm with $100 \%$ pixel fill-factor. The camera was cooled to − 90 °C using peltier and water cooling. Exposure time for each readout mode was set to the minimum. Chroma T4551pxt dichroic mirrors with a cutoff wavelength of $455 \text { nm}$ and $98 \%$ transmission at $710 \text { nm}$ were used to remove the pump beam. The tilt adjustable interference filter is a Chroma ET710/10m interference filter, with a $10 \text { nm}$ bandpass with a top-hat profile centred at $710 \text { nm}$ ($99 \%$ efficiency). The fixed interference filter positioned on the camera is a Semrock FF01-711/25 interference filter, with a $25 \text { nm}$ bandpass with a top-hat profile centred at $711 \text { nm}$ ($99 \%$ efficiency).

## Data Availability

Data required to evaluate the conclusions in this paper are available from the corresponding author upon reasonable request.
